# Does Twitter improve the dissemination of ophthalmology scientific publications?

**DOI:** 10.1007/s10792-023-02849-1

**Published:** 2023-08-12

**Authors:** Thomas Muecke, Stephen Bacchi, Robert J. Casson, WengOnn Chan

**Affiliations:** 1 Royal Adelaide Hospital, Adelaide, SA 5000 Australia; 2 University of Adelaide, Adelaide, SA 5005 Australia; 3 Flinders University, Bedford Park, SA 5042 Australia; 4 Ophthalmology Department, Royal Adelaide Hospital, Port Road, Adelaide, SA 5000 Australia

**Keywords:** Twitter, Machine learning, Academic citations, Perfomance metrics

## Abstract

**Purpose:**

To determine whether Twitter improves dissemination of ophthalmology scientific publications

**Methods:**

Data were collected on articles published on PubMed between the years 2016 and 2021 (inclusive) and identified with the word “ophthalmology”. Twitter performance metrics, including the number of tweets, number of likes, and number of retweets were collected from Twitter using the publicly available scientific API. Machine learning and descriptive statistics were used to outline Twitter performance metrics.

**Results:**

The number of included articles was 433710. The percentage of articles that were in the top quartile for citation count, which had ≥1 tweet was 34.4% (number 437/1270). Conversely, the percentage of articles that were in the top quartile for citation count, which had 0 tweets was 27.8% (number 12023/43244). When machine learning was used to predict Twitter performance metrics an AUROC of 0.78 was returned. This was associated with an accuracy of 0.97

**Conclusion:**

This study has shown preliminary evidence to support that Twitter may improve the dissemination of scientific ophthalmology publications.

Dear Editor,

Ophthalmic practitioners, researchers and journals (ie @JAMAOphth, @AAOJournal) use Twitter to share and discuss peer-reviewed scientific ophthalmology publications [1–4]. Users can react to posts containing ophthalmic research using likes, comments, and retweets, which “pushes” the original post to a new set of users. There is, however, limited evidence to demonstrate the efficacy of Twitter for improving the citation count of scientific ophthalmology publications. In the present study, we utilise machine learning techniques to (1) characterise the performance metrics associated with Twitter usage in relation to scientific ophthalmology publications, and (2) examine the effectiveness of machine learning in the prediction of these Twitter performance metrics.

Data were collected on articles published on PubMed between the years 2016 and 2021 (inclusive) and identified with the word “ophthalmology”. Twitter performance metrics, including the number of tweets, number of likes, and number of retweets were collected from Twitter using the publicly available scientific API. Tweets were included that used the term "pubmed.ncbi.nlm.nih.gov". Tweets were linked to individual articles with PubMed ID.

Descriptive statistics were used to outline Twitter performance metrics. Wilcoxon signed rank tests (due to the non-parametric nature of the data as evaluated with Shapiro–Wilk tests) and chi squared tests were used to compare article characteristics with respect to title length, abstract length, and number of authors. Data underwent pilot machine learning analysis to gauge whether a signal were present in the prediction of Twitter performance metrics. Namely, text (title, abstract, author list, journal ISSN, and MeSH terms for each article) underwent pre-processing (capitalisation removal, negation detection, word stemming, stop-word removal, and conversion to a term-frequency inverse document frequency table), and were then randomly split into training and testing datasets (75%/25%). A XGBoost was then developed on the training dataset, prior to evaluation on the holdout test dataset, aiming to predict whether any given article would receive ≥ 1 tweet or no tweets. This model was chosen due to the imbalanced nature of the dataset. The primary outcome was the area under the receiver operator curve (AUROC). This study did not require institutional ethical approval due to the use of publicly available data.

The number of included articles was 433,710. The number of articles which had ≥ 1 tweet identifiable was 1270 (0.29%) (see Table 1). Overall, the median citation count was 2 (IQR 0 to 6). For articles that had tweets, the median tweet count was 1 (IQR 1 to 2), the median cumulative like count was 1 (IQR 0 to 5), and the median cumulative retweets was 0 (IQR 0 to 2). The percentage of articles that were in the top quartile for citation count, which had ≥ 1 tweet was 34.4% (number 437/1270). Conversely, the percentage of articles that were in the top quartile for citation count, which had 0 tweets was 27.8% (number 12023/43244).

**Table 1:** Characteristics of the included articles

Characteristic	Articles with 0 tweets (*N* = 43,244)	Article with ≥ 1 tweet (*N* = 1270)	*P* value
Year median (IQR)	2019 (2017–2021)	2021 (2020–2021)	< 0.001
Title length median characters (IQR)	100.0 (76.0–126.0)	98.0 (75.0–125.0)	0.213
Abstract length median words (IQR)	13.0 (10.0–16.0)	13.0 (10.0–16.0)	0.756
Abstract length median characters (IQR)	1538.0 (1153.0–1800.0)	1556.0 (1128.0–1870.0)	0.013
Abstract length median words (IQR)	222.0 (165.0–259.0)	225.0 (159.0–267.0)	0.083
Author median number (IQR)	5.0 (4.0–8.0)	7.0 (4.0–10.0)	< 0.001
Citation count median (IQR)	2.0 (0.0–6.0)	3.0 (0.0–9.0)	< 0.001
Citations ≥ 2 (%)	24,457 (56.6)	772 (60.8)	0.003
Citations ≥ 6 (%)	12,023 (27.8)	437 (34.4)	< 0.001

When machine learning was used to predict Twitter performance metrics an AUROC of 0.78 was returned. This was associated with an accuracy of 0.97 (10 true positive, 345 false negative, 10,764 true negative, and 10 false positive). It can be seen that these performance characteristics need to be viewed in the context of an unbalanced test dataset.

This study has shown preliminary evidence to support that Twitter may improve the dissemination of scientific ophthalmology publications. We report that scientific ophthalmology publications with higher performance on Twitter metrics, namely having at least one tweet, may be associated with higher performance on conventional academic metrics such as citations. It is important to note the observational and retrospective nature of this analysis. We recgonise that the “black box” nature of machine learning lessens interpretability, and that reverse causality may account for the study findings by producing a biased model. Further studies may seek to trial engagement with social media in a randomised manner to gauge whether such activity influences the scientific impact of articles. Continued work in this field may also explore the effect of the dissemination of false or misleading ophthalmic research findings through Twitter, a topic of growing concern [5].
